# Incorporation of Au Nanoparticles on ZnO/ZnS Core Shell Nanostructures for UV Light/Hydrogen Gas Dual Sensing Enhancement

**DOI:** 10.3390/membranes11110903

**Published:** 2021-11-22

**Authors:** Yu-Sheng Tsai, Deng-Yi Wang, Jia-Jie Chang, Keng-Tien Liang, Ya-Hsuan Lin, Chih-Chen Kuo, Ssu-Han Lu, Yewchung Sermon Wu, Lukas Jyuhn-Hsiarn Lee, Hsiang Chen, Dong-Sing Wuu

**Affiliations:** 1 Department of Materials Science and Engineering, National Yang Ming Chiao Tung University, Hsinchu 300, Taiwan; s0781509.mse07g@nctu.edu.tw (Y.-S.T.); sermonwu@faculty.nctu.edu.tw (Y.S.W.); 2 Department of Applied Materials and Optoelectronic Engineering, College of Science and Technology, National Chi Nan University, Puli 545, Taiwan; s109328505@mail1.ncnu.edu.tw (D.-Y.W.); s109328502@mail1.ncnu.edu.tw (J.-J.C.); s108328016@mail1.ncnu.edu.tw (K.-T.L.); s108328042@mail1.ncnu.edu.tw (Y.-H.L.); s107328003@mail1.ncnu.edu.tw (C.-C.K.); s106328016@mail1.ncnu.edu.tw (S.-H.L.); dsw@ncnu.edu.tw (D.-S.W.); 3 National Institute of Environmental Health Sciences, National Health Research Institutes, Miaoli 350, Taiwan; 4 Stroke Center, Departments of Neurology, Environmental and Occupational Medicine, National Taiwan University Hospital, Taipei 100, Taiwan; 5 Institute of Environmental and Occupational Health Sciences, College of Public Health, National Taiwan University, Taipei 100, Taiwan; 6 Research Center for Environmental Medicine, Ph.D. Program of Environmental and Occupational Medicine, College of Medicine, Kaohsiung Medical University, Kaohsiung 807, Taiwan

**Keywords:** ZnO/ZnS core shell, Au nanoparticle, dual sensing, hydrogen, UV light, electric field

## Abstract

ZnO/ZnS nanocomposite-based nanostructures exhibit dual light and gas sensing capabilities. To further boost the light/dual sensing properties, gold nanoparticles (Au NPs) were incorporated into the core-shell structures. Multiple material characterizations revealed that Au NPs were successfully well spread and decorated on ZnO/ZnS nanostructures. Furthermore, our findings show that the addition of Au NPs could enhance both 365 nm UV light sensing and hydrogen gas sensing in terms of light/gas sensitivity and light/gas response time. We postulate that the optimization of gas/light dual sensing capability may result from the induced electric field and inhabitation of electron-hole recombination. Owing to their compact size, simple fabrication, and stable response, ZnO/ZnS/Au NPs-based light/gas dual sensors are promising for future extreme environmental monitoring.

## 1. Introduction

With the advancement of nanotechnology, various ZnO-based nanocomposite shapes have been fabricated [1]. Among these nanocomposites, ZnO/ZnS core-shell structures have drawn growing attention because of their distinct optical and material properties [2,3,4,5]. Recently, ZnO/ZnS core-shells have been used as photocatalysts [6], electrochemical devices [7], solar fuels [8,9], and sensors. In order to further boost ZnO/ZnS functional properties, various additives such as Cr [10], Ni [11], and Au [12] have been incorporated into the nanocomposites. In 2021, ZnO/ZnS core-shell nanomaterials were demonstrated for use as UV light/CO gas dual sensors [13]. However, over the past decade, only a few light/gas dual sensing devices have been reported. In addition to ZnO-based nanocomposites, ZnS and In-Ga-Zn-O materials have been used for light/gas dual sensing devices. Comparisons of some recently demonstrated light/gas dual sensors and those found in this work are listed in Table 1 [13,14,15,16]. Based on a previous study [12], Au nanoparticles (NPs) have been added to hollow ZnO/ZnS core-shells to enhance photocatalytic hydrogen evolution. Since the addition of Au NPs can inhibit the recombination of electron hole pairs, incorporating Au NPs can possibly enhance light/gas dual sensing properties because the strengthening of electron hole pair generation can foster the increase of the light and gas-induced current. Until now, the addition of gold (Au) nanoparticles (NPs) onto ZnO nanocomposites for dual sensing enhancement has not been clearly reported. It is therefore worthwhile to explore novel treatments, new processes, and alternative material combinations. In this study, Au NPs were added to ZnO/ZnS core-shell nanostructures to optimize the material properties and sensing applications [17,18,19]. A new nanocomposite with the addition of Au NPs could further enhance UV light/hydrogen gas dual sensitivity and shorten the gas/light response/recovery time owing to electrical and optical enhancements. The detection of skin cancer-causing UV light [17] and monitoring of inflammable hydrogen gas [18,19] are crucial to environmental safety. Owing to their compact size, simple fabrication, and good sensing performance, ZnO/ZnS/Au NPs-based UV light/hydrogen gas dual sensors are promising for future environmental and detectional use.

## 2. Materials and Methods

This experiment used silicon dioxide substrate with a lattice direction of <100> p-type and an oxide layer of 100 nm.

**Figure 1:**
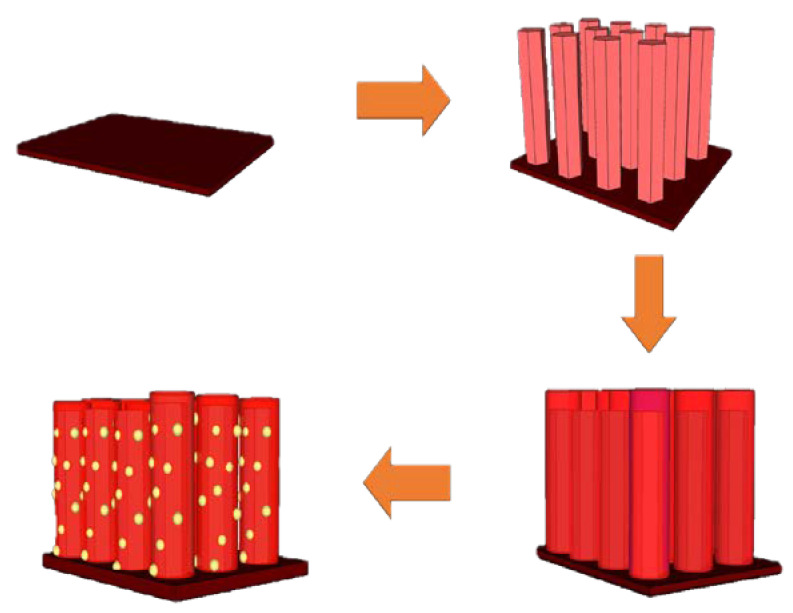
Schematic diagram of the manufacturing process of ZnO/ZnS/Au NPs on SiO_2_ substrates.

A 6-inch silicon wafer with a wet oxide thickness of 1 μm was purchased from the Wellbeing Co, HsinChu, Taiwan. The wafer was cut into 1 cm × 1 cm substrates. These substrates were first cleansed with alcohol for 5 min, acetone and isopropanol were then used to wash the substrates. The silicon dioxide substrate was then used to grow ZnO nanorods (NRs) and ZnS was formed on ZnO NRs in an ion exchanging reaction. Finally, Au NPs were dropped onto the surface of the ZnS shell. Figure 1 illustrates the manufacturing process of ZnO/ZnS/Au NPs.

### 2.1. Preparation of Sensor Chip Substrate

To fabricate ZnO sensor chips, p-type substrates were first cleansed with isopropyl alcohol, acetone, and deionized water. Then, a dioxide of 1 μm thickness was thermally grown on the silicon substrate in a furnace at 1000 °C. This was followed by the deposit of a SiO_2_ layer, after which the patterns of integer-fingers contact, electrodes were defined using photolithography. The electrodes were formed by lift-off after an Au/Cr metal layer was grown. The stacked Au/Cr layer was deposited on the front of the SiO_2_ layer by E-gun evaporation. A 200 nm Au layer, with a thin Cr layer between the Au layer and the SiO_2_ layer, was evaporated. The adherence between the Au and the SiO_2_ was improved by inserting a Cr layer.

### 2.2. Preparation of the ZnO Seed Layer

The seed layer was prepared using the sol-gel method. Zinc acetate [Zn(CH_3_COO)_2_, 0.05 M] was mixed with 60 mL of ethanol [C_2_H_5_OH], and 2 drops of monoethanolamine (MEA) were added as a stabilizer in a spin coating solution. The SiO_2_ substrate was placed in a spin coater, and then the spin coating liquid was dropped onto the surface. A 50 μL pipette was used to spread the substrate surface (one drop for 50 μL). The spin coating was 500 rpm for 5 s and then 3000 rpm for 30 s. The spin coating treatment was repeated 5 times.

### 2.3. Preparation of ZnO NRs

A hydrothermal method was used to grow ZnO NRs. Hexamethylenetetramine (HMT) and zinc nitrate [Zn(NO_3_)_2_] were added to deionized water as a growth solution. The substrate was fixed on the glass slide and put into a serum bottle containing the growth solution. The temperature for the hydrothermal growth of ZnO was 80 °C and the growth time was 1 h [20].

### 2.4. Synthesis of ZnS Shells on the Surface of ZnO NRs

A hydrothermal method was used to synthesize zinc sulfide [21]. ZnO was used as the reaction template for anion exchange to synthesize zinc sulfide. Sodium sulfide was added to deionized water as a growth solution. Similarly, the substrate was put on a glass slide and put into the serum bottle containing the growth solution. The temperature of hydrothermal growth for ZnO was 70 °C and the growth time was 10 min.

### 2.5. 20 nm Au NP Dropping

The 20 nm Au NPs (Au NPs: 20 nm Ted Pella Inc., Hsinchu, Taiwan. Prod No. 15705-20) were used to decorate the ZnO/ZnS core-shell structures. A solution containing 20 μL of Au NPs was dropped on the substrate and dried on the heating plate at 130 °C. These steps were repeated 3 times.

### 2.6. Gas Sensitivity

To evaluate hydrogen gas sensing capability, the sample was placed in a chamber and two electrodes were linked to a Keithley 237 Source-Measure Unit. A bias voltage of 5 V was applied between the two electrodes and the resistance was measured. After the chamber was heated to 300 °C and the concentration of hydrogen gas was 300 ppm, gas sensitivity could be evaluated by the variation of the induced current between the two electrodes, as shown in Equation (1):

S% = [(I_gas_ − I_air_)/I_air_] × 100 (1)

I_gas_: With measuring gas (H_2_/CO)

I_air_: Zero air gas

I_gas_: 300 ppm (time per current for sampling)

I_air_: 300 ppm (time per current for sampling)

(I, current unit: mA)

### 2.7. Light Sensitivity

UVA light (wavelength = 365 nm) illumination light sensing measurements were conducted in a dark room. Alternation of 30 s of illumination and 30 s without illumination was performed and photo sensing was measured on the devices. The photon sensitivity was recorded by the change of the induced current, as shown in Equation (2):

S% = [(I_light_ − I_dark_)/I_dark_] × 100 (2)

I_light_: The current on the light

I_dark_: The current in the dark

I_light_: λ = 365 nm, (time per current for sampling)

(I, current unit: mA)

### 2.8. Material Characterizations

To examine ZnO/ZnS/Au NPs nanocomposites, multiple material characterizations, including field emission scanning electron microscopy (FESEM), energy-dispersive X-ray spectroscopy (EDX), transmission electron microscope (TEM), X-ray photoelectron spec-troscopy (XPS), photoluminescence (PL), and X-ray diffraction (XRD) were performed. Results indicate that Au NPs were successfully spread on ZnO/ZnS nanostructures. The ZnO/ZnS/Au NPs structures were also grown on sensor chips to function as dual sensing devices as the incorporation of Au NPs could generate a high electric field and hence speed up the carrier movement [22].

(1) FESEM and TEM: The FESEM and TEM images were obtained using JEOL JSM-7500F and JEOL JEM 2100 PLUS instruments, respectively. The operating voltages for SEM and TEM were 15 kV and 200 KV, respectively.
(2) XPS: The XPS instrument was a VG Scientific ESCALAB 250 spectrometer. The light source was a twin anode X-ray gun with a maximum energy of 15 kV. The XR5 mono-chromated X-ray gun, with a maximum energy of 15 kV, 200 W, had an aluminum target with a beam size of 650–120 um.
(3) PL: The PL spectra were obtained using a HI-TACHI F-4500 fluorescence spectrophotometer. The excitation laser wavelength was 325 nm with a laser spot diameter of 1 μm. The PL spectral range was 330–1000 nm (CCD sensor) and 1000–1500 nm (In-GaAs sensor).
(4) XRD: The XRD patterns were acquired using a Bruker D8 Discover microdiffractometer. For the XRD analysis of the samples, a grazing incidence of X-ray beam CuKa (k = 1.542 Å) radiation was used with an incidence angle step of 0.5° in the diffraction angle range (2θ) from 20° to 60°.

## 3. Results and Discussion

**Figure 2:**
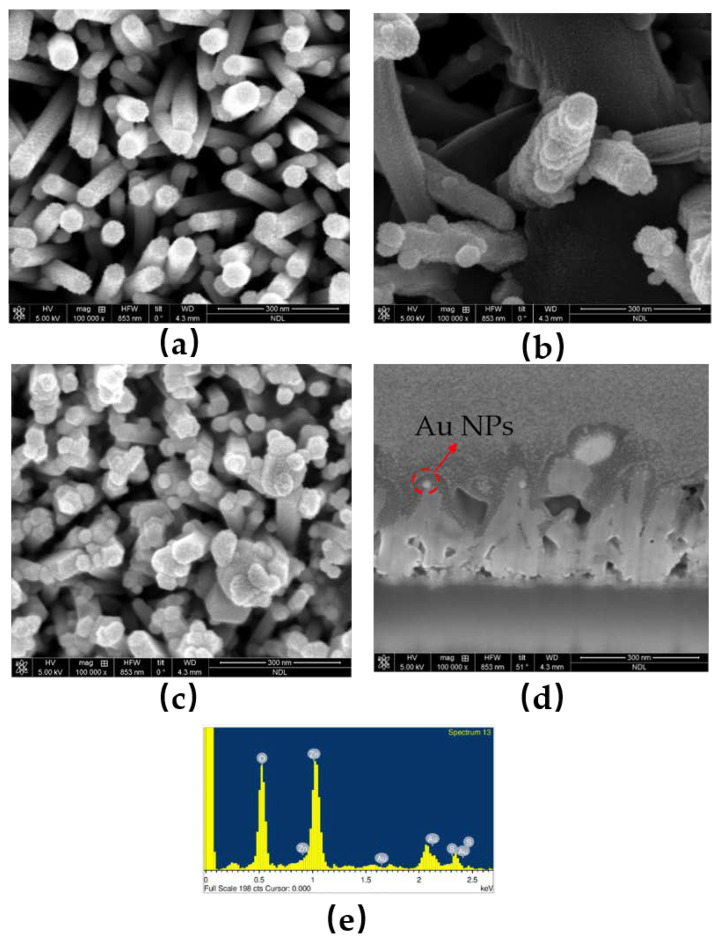
(**a**) An FESEM image of ZnO nanorods; (**b**,**c**) FESEM images of ZnO/ZnS/Au NPs nanocomposites; (**d**) A cross-section SEM image of ZnO/ZnS/Au NPs. (**e**) EDX analysis of ZnO/ZnS/Au NRs.

To examine the surface morphologies of ZnO NRs and ZnO/ZnS/Au NPs nanocomposites, FESEM analysis was used. As shown in Figure 2a, smooth surfaces could be observed on ZnO NRs. After the ZnS shells were grown on the ZnO NRs, fluffy surfaces could be seen, as shown in Figure 2b,c. Furthermore, small Au NPs were sparsely distributed on ZnO/ZnS NRs. To zoom in on the randomly distributed Au NPs, a cross-section image, as shown in Figure 2d, reveals that Au NPs, as indicated by the red arrows, had sunk in and were trapped in the ZnS shell located near the NRs [22].

**Figure 3:**
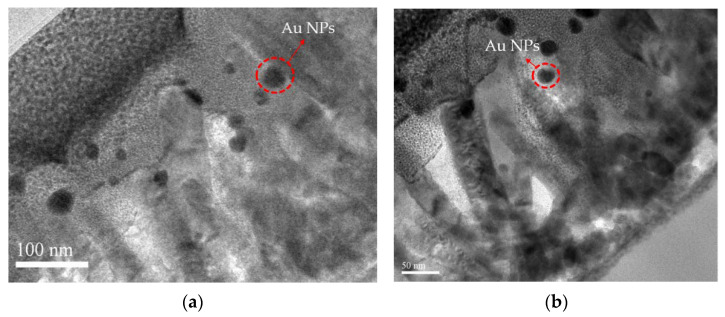
(**a**,**b**) TEM images of ZnO/ZnS/Au NPs nanocomposites in different locations.

To confirm the presence of the ZnS shell and Au NPs, the EDX analysis is shown in Figure 2e and the element compositions are shown in Table 2. Strong O, S, and Zn signals could be detected, indicative of the successful growth of ZnO/ZnS core-shell structures. Furthermore, 4.47% of Au, as shown in EDS analysis table of Table 2, reveals that the presence and sparse distribution of Au NPs are consistent with the FESEM images [23,24]. In addition, TEM images were taken to zoom in on the fine nanostructure of the nanocomposites based on ZnO/ZnS/Au NPs, TEM images were taken as shown in Figure 3a,b [25], and Au NPs are more clearly displayed. Moreover, Au NPs were randomly distributed and trapped in the ZnS shells near the ZnO NRs. In addition, Au NPs were located around the top of these ZnO/ZnS NRs, and were consistent with the cross-section FESEM image of Figure 2d.

**Figure 4:**
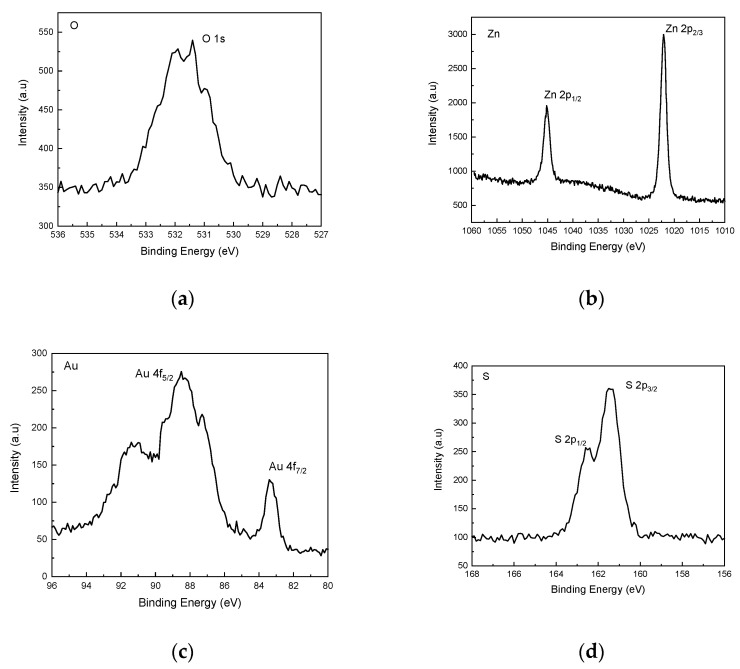
(**a**) The O 1s (**b**) Zn 2p (**c**) S 2p, and (**d**) Au 4f XPS spectra of the nanocomposite based on ZnO/ZnS/Au NPs.

In order to study the binding energies of the nanocomposite, the O 1s, Zn 2p, S 2p, and Au 4f XPS spectra were measured, as shown in Figure 4a–d. In comparison to the ZnO/ZnS core shell structures [26], Au NPs have lower binding energies for O 1s, Zn 2p, and S 2p since incorporating Au NPs may weaken the binding energies [26,27]. In addition, Au 4f exhibited a three-peak profile for the Au NPs, as shown in Figure 4d.

**Figure 5:**
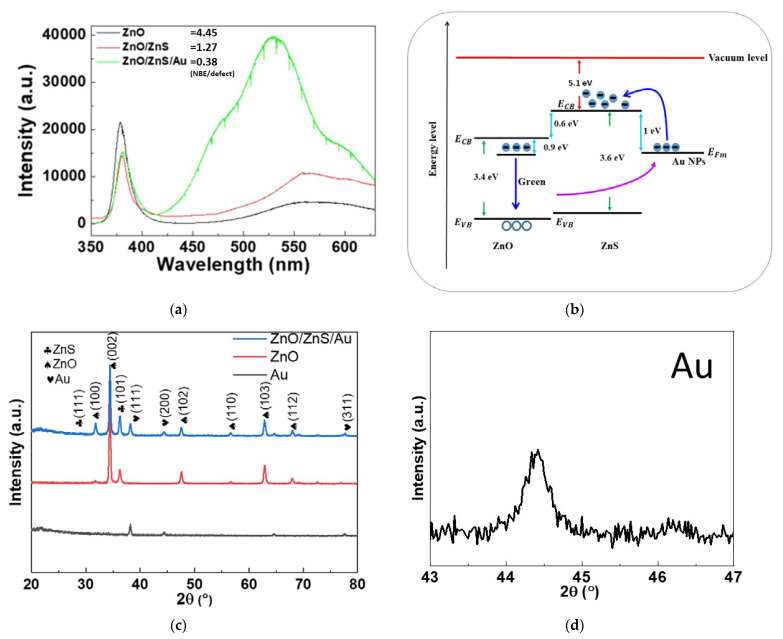

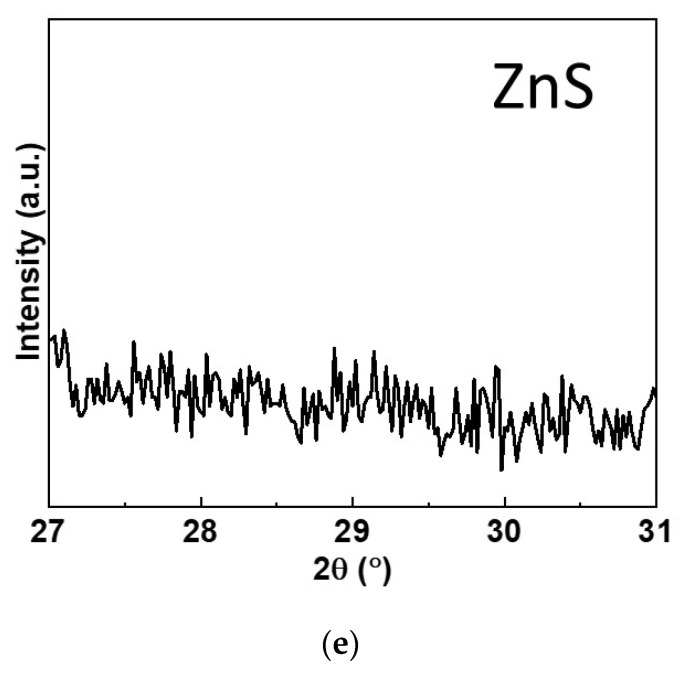
(**a**) PL measurements of ZnO NRs, ZnO/ZnS NRs, and ZnO/ZnS/Au NP nanocomposites; (**b**) The mechanism of defect luminescence enhancement by Au NPs (**c**) XRD patterns of ZnO NRs and ZnO/ZnS/Au NPs. The XRD peak of ZnO/ZnS/Au: Au (**d**) and ZnS (**e**).

In addition, PL measurements and XRD analysis were performed to investigate the optical properties and the crystalline structures, as shown in Figure 5. PL measurements show the near-band-edge (NBE) emission at around 375 nm and the defect-luminescence above 520 nm [28,29]. The PL spectrum of the pure ZnO NRs shows a high signal/noise ratio of the NBE/defect luminescence compared with ZnO/ZnS and ZnO/ZnS/Au NPs [30], as shown in Figure 5a. Specifically, the addition of Au could drastically enhance defect luminescence because strong electromagnetic (EM) fields around Au NPs facilitate transitions between the oxygen vacancy defects and the valence band, as shown in Figure 5b. Therefore, after the ZnS shell was grown and the Au NPs were dropped onto the NRs, the defect luminescence became much stronger and the NBE emission became weaker. Moreover, the strong EM field also sped up the carrier movement for gas/light sensing and increased the sensing capability. In addition, a red shift of the NBE peak, and the blue shift of the defect luminescence, can be observed. Based on a previous report [31], incorporation of ZnS and Au NPs may cause a heavy decorating effect, which induces band narrowing and increases the defect luminescence. XRD patterns, as shown in Figure 5c, present a noticeable wurtzite (111) ZnS peak and a strong (111) Au peak, which further strengthened the evidence of the growth of ZnS shells and the addition of Au NPs [32].

**Figure 6:**
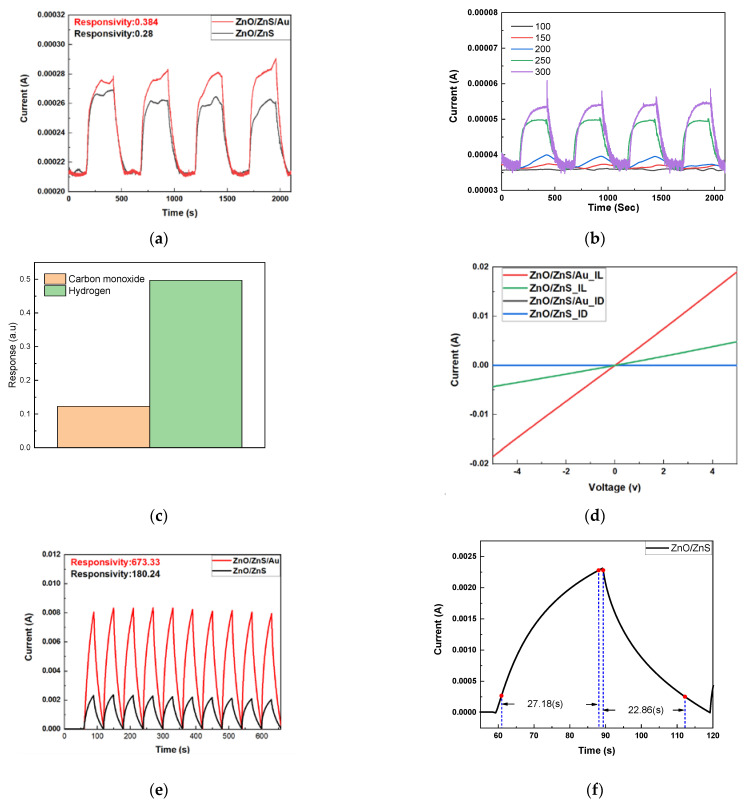

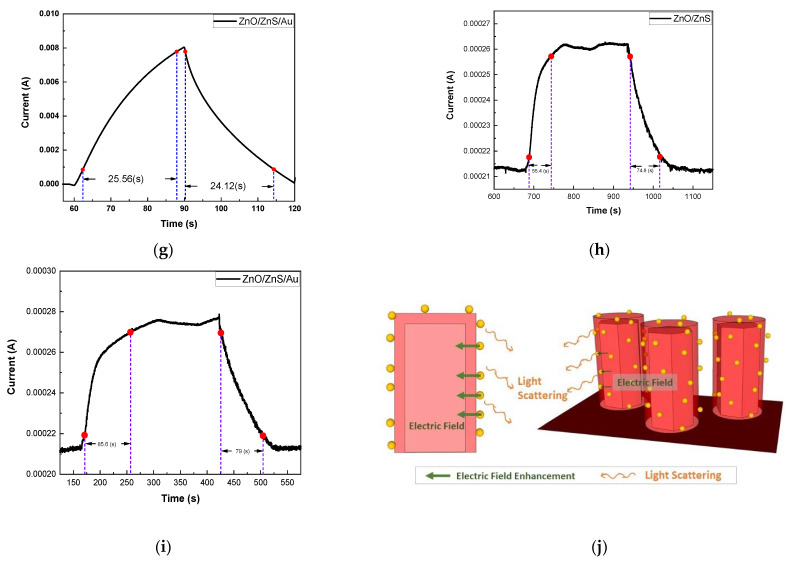
(**a**) Hydrogen gas sensing behaviors of ZnO/ZnS and ZnO/ZnS/Au NPs. (**b**) Hydrogen gas sensing behaviors of ZnO/ZnS/Au NPs at 100, 150, 200, 250, and 300 °C. (**c**) Selectivity between CO and H_2_ gas. (**d**) IV curves of ZnO/ZnS and ZnO/ZnS with Au NPs (I_D_: Dark current and I_L_: Light current). (**e**) UV sensing behaviors of the samples. The light sensing rise and recovery times of (**f**) ZnO/ZnS and (**g**) ZnO/ZnS with Au NPs. The gas sensing rise and recovery times of (**h**) ZnO/ZnS and (**i**) ZnO/ZnS with Au NPs. (**j**) The detailed mechanisms of dual sensing enhancement.

Finally, light/gas dual sensing measurements were performed on the various samples. The gas responsivity was calculated from the equation of R_G_ = (I_gas_ − I_air_)/I_air_, where I_gas_ is the current in ambient hydrogen and I_air_ is the current in ambient dry air. As shown in Figure 6a, noticeable improvements of hydrogen gas sensing could be observed for ZnO/ZnS/Au with the addition of Au NP compared with ZnO/ZnS. Based on a previous study [33], the increase of an electric field by depositing a ZnS shell to form a ZnS/ZnO interface could speed up carrier movement causing ZnO/ZnS gas sensors performed better than ZnO gas sensors. Similarly, the incorporation of Au NPs could enhance the electric fields around Au NPs and therefore further improve the gas sensing capability [34,35,36]. In addition, the hydrogen gas sensing behaviors of ZnO/ZnS/Au NPs at 100, 150, 200, 250, and 300 °C is shown in Figure 6b. Results indicate that as the ambient temperatures reached 250 °C, the gas sensitivity was greatly enhanced. Moreover, to present selectivity between CO and H_2_, a bar graph, as shown in Figure 6c, clearly shows that H_2_ sensitivity was much better than CO sensitivity [17]. In addition, UV light-sensing measurements were conducted for the samples, as shown in Figure 6d,e. The I-V curves of ZnO/ZnS and ZnO/ZnS/Au NPs are shown in Figure 6d. With the dark currents (I_D_) in both cases close to zero, the light current of the sample with an Au NP addition was much larger than the sample without Au NP addition. Furthermore, the photo pulse responsivity, calculated from the equation R_P_ = ΔI/I_D_, where ΔI was the difference between the photocurrent and I_D_ as the dark current, is shown in Figure 6e. This results also indicate that UV light sensing was greatly improved after the addition of Au NPs. Additionally, the light sensing rise and recovery times of ZnO/ZnS and ZnO/ZnS with Au NPs are shown in Figure 6f,g. Results indicate that the addition of Au NPs can effectively shorten the rise and fall times. Similarly, the gas sensing rise and recovery times of ZnO/ZnS and ZnO/ZnS with Au NPs are shown in Figure 6h,i. The conduction mechanism in the oxygen vacancy model is described as follows: (1) The gas consumes a lattice oxygen atom from the surface, producing an oxygen vacancy; (2) the vacancy becomes ionized, which introduces free elections to the conduction band, increasing the conductance of the n-type ZnO; and (3) an oxygen atom in the gas phase fills the vacancy, taking electrons from the conduction band, which decreases the conductivity [37].

The addition of Au NPs could improve dual sensing behaviors both in terms of response time and sensitivity. In addition to speeding up the carrier mobility, Au NPs might scatter the light and strengthen light absorption [38]. The detailed mechanisms of electric field enhancement and light scattering are shown in Figure 6e. Light absorption at the nanorod’s surface can capture the free electron present in the n-type semiconductor, forming a low-conductivity depletion layer near the surface. As a high work function metal, Au forms a localized Schottky barrier in the vicinity of Au NPs, which increases the height and the width of space chare region [39]. Therefore, UV light sensing capacity can be drastically improved.

## 4. Conclusions

In this study, nanocomposites based on ZnO/ZnS/Au NPs were fabricated. Morphological measurements including FESEM and TEM indicate that ZnS shells could be grown on the ZnO NRs and Au NPs could be well-distributed in the ZnS shells. XPS, EDX, and XRD patterns confirmed the presence of ZnS shells and Au NPs. Multiple optical and material analyses indicate that Au incorporation can cause light scattering and electric field enhancement. Therefore, based on the light and gas sensing measurements, the light/gas dual sensitivity were increased, and light/gas sensing rise/recovery times were shortened. Results indicate that the ZnO/ZnS/Au NPs photo sensor chip had much better UV light sensing performance than ZnO/ZnS NRs due to light scattering caused by the Au NPs. This scattering effect could enhance light absorption and greatly strengthen UV sensing. In addition, the ZnO/ZnS/Au NPs hydrogen gas sensor chip had better gas sensing performance than ZnO/ZnS NRs because of the strengthening of electric fields induced by Au NPs. Electric field enhancement could increase the carrier transition speed and shorten the gas sensing rise/recovery time. Owing to their compact size, simple fabrication, and stable performance, nanocomposites based on ZnO/ZnS/Au NPs light/gas dualsensing devices are promising for future environmental monitoring technology.

## Figures and Tables

**Table 1:** Reported semiconductor-based gas/light dual sensors.

Item	Materials	Gas/Light	Operating Codition	Sensivity	Ref.
1	ZnS	Gas: Acetone, Ethanol Light: 254 (nm), 365 (nm)	Gas: 320 °C Light: 5 V	Gas: 21.1 ^a^, 13.3 ^a^ Light: 12 ^b^, 28 ^b^	[14]
2	In-Ga-Zn-O	Gas: O_3_ Light: 365 (nm)	Gas: UV intensity ~945 mW/m^2^ Light: N/A	Gas 4.74 ^c^ Light: 23,924.31 ^d^	[15]
3	ZnO/ZnS	Gas: CO Light: 395 (nm)	Gas: 200 °C Light: 0.1 V	Gas 1.561 ^e^ Light: 65.6 ^f^	[13]
4	ZnO/ Perylene diimide	Gas: CO Light: 395 (nm)	Gas: 200 °C Light: 2 V	Gas 1.0851 N/A Light: 4.114 (A/W)	[16]
5	ZnO/ZnS/Au	Gas: H_2_ Light: 365 (nm)	Gas: 300 °C Light: 5 V	Gas 0.384 ^c^ Light: 673.33 ^d^	This work

^a^ The sensitivity (S) defined as S = R_a_/R_g_, where R_a_ is the sensor resistance in air and R_g_ is the resistance (unit: Ω) in the target atmosphere. ^b^ S = I_Light_/I_Dark_. ^c^ S = (R_a_ − R_g_)/R_a_. ^d^ S = (I_Light_ − I_Dark_)/I_Dark_. ^e^ S% = [(R_a_ − R_g_)/R_a_] × 100%. ^f^ S% = [(I_Light_ − I_Dark_)/I_Dark_] × 100%.

**Table 2:** EDX analysis data of ZnO/ZnS/Au NPs.

	O	S	Zn	Au
Weight%	32.08	13.04	50.41	4.47
Atomic%	62.55	12.69	24.05	0.71
